# Comparison of Two-Talker Attention Decoding from EEG with Nonlinear Neural Networks and Linear Methods

**DOI:** 10.1038/s41598-019-47795-0

**Published:** 2019-08-08

**Authors:** Gregory Ciccarelli, Michael Nolan, Joseph Perricone, Paul T. Calamia, Stephanie Haro, James O’Sullivan, Nima Mesgarani, Thomas F. Quatieri, Christopher J. Smalt

**Affiliations:** 1 Bioengineering Systems and Technologies Group, MIT Lincoln Laboratory, Lexington, MA USA; 2 Speech and Hearing Bioscience and Technology, Harvard Medical School, Boston, MA USA; 3 Department of Electrical Engineering, Columbia University, New York, NY USA

**Keywords:** Translational research, Biomedical engineering, Auditory system

## Abstract

Auditory attention decoding (AAD) through a brain-computer interface has had a flowering of developments since it was first introduced by Mesgarani and Chang (2012) using electrocorticograph recordings. AAD has been pursued for its potential application to hearing-aid design in which an attention-guided algorithm selects, from multiple competing acoustic sources, which should be enhanced for the listener and which should be suppressed. Traditionally, researchers have separated the AAD problem into two stages: reconstruction of a representation of the attended audio from neural signals, followed by determining the similarity between the candidate audio streams and the reconstruction. Here, we compare the traditional two-stage approach with a novel neural-network architecture that subsumes the explicit similarity step. We compare this new architecture against linear and non-linear (neural-network) baselines using both wet and dry electroencephalogram (EEG) systems. Our results indicate that the new architecture outperforms the baseline linear stimulus-reconstruction method, improving decoding accuracy from 66% to 81% using wet EEG and from 59% to 87% for dry EEG. Also of note was the finding that the dry EEG system can deliver comparable or even better results than the wet, despite the latter having one third as many EEG channels as the former. The 11-subject, wet-electrode AAD dataset for two competing, co-located talkers, the 11-subject, dry-electrode AAD dataset, and our software are available for further validation, experimentation, and modification.

## Introduction

Hearing loss, and the associated use of hearing-aids, is rising among the general population^1^, and as shown by recent statistics from the US Dept. of Veterans Affairs, is particularly prevalent among retired military personnel^2^. Despite widespread use of hearing aids, and the incorporation of spatial and spectral algorithms for noise reduction, hearing-aids often are considered unsatisfactory in regard to their performance in noisy environments^3–5^. Particularly when background noise includes other talkers, hearing aids suffer because they have difficulty separating the “signal” (*i*.*e*., the talker of interest to the listener) from the “noise” (*i*.*e*., all other talkers) due to similarities in spectro-temporal characteristics. The failure of hearing aids to improve listening ability in complex acoustic environments, either due to poor device performance, or lack of use triggered by poor performance, is associated with social isolation and various forms of cognitive decline such as depression^6–8^. Therefore, solving the problem of assisted listening in multi-talker environments could have wide societal benefits in terms of communication and mental health. Auditory attention decoding (AAD) is a recent approach aimed at such a solution, one which exploits knowledge of the listener’s auditory intent (attention) to isolate and enhance the desired audio stream and suppress others.

Evidence for neural encoding of speech has been shown with various sensing modalities including electroencephalography (EEG)^9^, magnetoencephalography (MEG)^10^, and electrocorticography (ECoG)^11^. The exploitation of such encoding for AAD in a two-talker paradigm was initially demonstrated by Mesgarani and Chang^12^, through a classifier acting on speech spectrograms reconstructed from ECoG data. Comparison of the predicted spectrograms with those from the actual speech sources provided the identity of the attended talker with 93% accuracy when the subjects were known to be attending to the instructed stimulus. Since then, AAD has been achieved successfully with many variations on this initial technique^13–24^.

**Figure 1:**
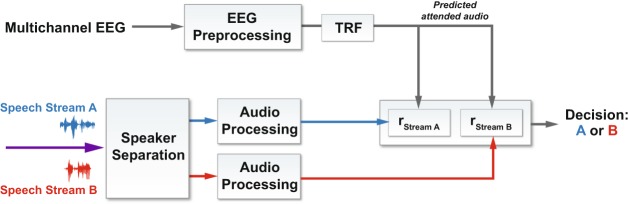
System architecture for auditory attention decoding: backward model. The temporal response function (TRF) can be linear or non-linear (neural network, see Fig. 3).

The most common approach to AAD, first described in O’Sullivan *et al*.^13^ and depicted in Fig. 1, involves EEG for capturing neural data as a more practical and less invasive modality than ECoG. The approach uses a linear least-squares method for stimulus (broadband speech envelope) reconstruction and correlation of actual and predicted speech envelopes to identify the attended talker. Stimulus reconstruction is also known as the “backward” problem in AAD, as the mapping from EEG to stimulus is the reverse of the natural auditory stimulus/response phenomenon. By contrast, predicting EEG from the stimulus is known as the “forward” problem.

The attention decision typically is between two simultaneous, spatially separated talkers. This approach has been modified to evaluate: sensitivity to number of EEG channels and size of training data^14^; robustness to noisy reference stimuli^15,16^; the use of auditory-inspired stimulus pre-processing including subband envelopes with amplitude compression^17^; cepstral processing of EEG and speech signals for improved correlations^25^; the effects of speaker (spatial) separation and additional speech-like background noise^18^; the effects of (simulated) reverberation^19^; and potential performance improvements through various regularization methods^20^.

Considering the AAD pipeline as comprising steps for neural data acquisition, stimulus representation, signal processing (*e*.*g*., forward or backward predictive modeling), and attention determination, alternate techniques have been described with variations of each of these components. MEG^26^ and ECoG^27^ continue to serve as neural sensing modalities, while EEG channels have been reduced in number in an effort to move toward less obtrusive, portable systems^21,22^. Speech stimuli have been represented with spectrograms^27^ and frequency-dependent envelopes after gammatone filtering^28^. To exploit the power and biological relevance of non-linear processing, effective implementations of the backward model with neural networks have been shown^23^, and while much less popular, linear versions of the forward model (predicting EEG from the stimuli) are described in Fiedler *et al*.^22^ and Wong *et al*.^20^. As an alternative to both forward and backward modeling, canonical correlation analysis, which involves transforming both stimulus and response to maximize mutual projections and thus improve correlations, has been applied to EEG and audio data, both with various filters, to enhance AAD performance^29^. Finally, state-space models have been applied as a final step in AAD systems to smooth noisy attention decisions and allow for near real-time update rates^24^.

Measuring the performance of AAD systems typically involves an intuitive computation of decoding accuracy, *i*.*e*., the percentage of decoding opportunities for which the system correctly identifies the attended talker. Overall results often are generated with a leave-one-out cross-validation scheme iterated over the collected dataset. This approach is used in both the backward^13–15,17,20^ and forward^22^ modeling paradigms. System accuracy also has been reported for predicting the *unattended* talker^13,19^, but in both cases performance is worse than that for predicting the attended talker. In Miran *et al*.^24^, the ${\ell }_{1}$-norm of the attended and unattended decoder coefficients are used as “attention markers” to generate a smooth, near real-time (~2-second latency) attentional probability through a state-space estimator. Talker classification is considered correct if the probability estimate *and* its 90% confidence interval for the attended talker are above 0.5, and accuracy is again measured as the percentage of correctly classified opportunities. In de Taillez *et al*.^23^ and Wong *et al*.^20^, performance is reported as an information transfer rate, *i*.*e*., the number of correct decoding decisions per minute.

**Figure 2:**
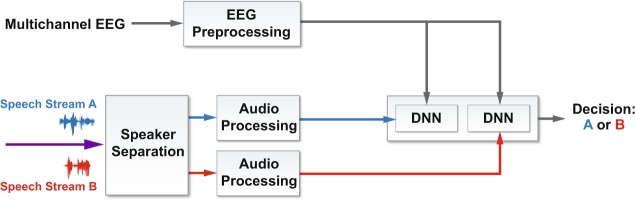
System architecture for auditory attention decoding: DNN binary classification. See Fig. 4 for a specific instance of the DNN.

Comparison of performance statistics across different published results, even those using the same decoding approach and performance metric, is hampered by variations in experimental parameters including talker number, angular separation, and gender, as well as number/placement of EEG electrodes, and by variations in processing parameters such as EEG or speech-envelope bandwidths, and correlation lags and window sizes. To address these barriers, in this paper we describe two datasets and three decoding algorithms along with results from each of the six combinations. The datasets include wet and dry EEG data collected from 11 subjects during an auditory-attention experiment with two simultaneous, co-located talkers (one female, one male). The algorithms include a linear stimulus-reconstruction decoder described in O’Sullivan *et al*.^13^ and a neural-network stimulus-reconstruction decoder described in de Taillez *et al*.^23^, and we introduce a novel convolutional neural-network classifier that predicts the attended talker without explicit stimulus or response prediction (Fig. 2). Our results indicate that this new architecture outperforms the traditional stimulus-reconstruction decoders by a significant margin on both datasets.

## Methods

### AAD experimental collection

#### Protocol

Speech from two co-located talkers, one male, and one female, was presented to each subject in a quiet, electrically shielded audiometric booth. The audio was presented from a single loudspeaker directly in front of the subject, with the experiment lasting approximately 40 minutes. The stimuli consisted of four “wikiHow.com” instructions lasting approximately 5 minutes each: “How to Make Waffles”, “How to Take Care of a Dog”, “How to be a Shepherd”, and “How to Identify Birds”. Each story (attended audio) was heard twice, once read by the male and once by the female talker, with a different story by the opposite gender presented simultaneously as the distractor (unattended) audio stream. The order of the two talkers, as well as the attended and distractor audio streams were randomized for each subject. Participants were instructed as to which gender talker to focus on at the start of each story on a screen in front of them throughout the experiment. Each story was interrupted randomly after 5–10 sentences were presented, and the participant was asked to repeat the last sentence of the attended talker. We term each uninterrupted listening interval as a “part”. A subset of subjects also participated in an auditory oddball task, but that data is not part of this analysis.

#### Subjects

The experimental protocol was approved by the MIT Committee on the Use of Humans as Experimental Subjects and the US Army Medical Research and Materiel Command (USAMRMC) Human Research Protection Office. All research was conducted in accordance with the relevant guidelines and regulations for human subject testing required by these committees. All subjects gave written informed consent to participate in the protocol.

Eleven MIT Lincoln Laboratory employees (7 male, 4 female) agreed to participate in two experiments on different days. The first experiment used a wet EEG system, and the second used a dry EEG system. All participants self-reported normal threshold hearing.

To ensure that subjects were on task, as well as potentially to exclude subjects that were unwilling or unable to attend to the target speaker, we checked the randomized interruptions of the stimuli presentations for a qualitative measure of attention. No subjects were excluded due to performance concerns. Several of the authors did participate in the study.

#### EEG instrumentation and preprocessing

Wet electrode EEG data were collected using a Neuroscan 64-channel Quik-Cap and a SynAmps RT amplifier with a sampling rate of 1000 Hz, and recorded in Curry data-acquisition software (Compumedics, Charlotte, NC) with a high-pass cut-off of 0.05 Hz and a low-pass cut-off of 400 Hz. Additional electrodes were placed on both mastoids, as well as for some subjects above, below, and next to the left eye. The reference electrode was located halfway between CZ and CPZ. Dry electrode EEG data were collected using a Wearable Sensing DSI-24 system (San Diego, CA), a joint sensor platform and signal amplifier. The system records from 18 scalp channels and two reference channels attached to the subject’s earlobes. Data were collected at a 300 Hz sampling rate using DSI-Streamer software, with a high-pass cut-off of 1 Hz and low-pass cut-off of 50 Hz.

Prior to analysis, all EEG data were down-sampled to 100 Hz using MATLAB’s resample function (Mathworks, Natick, MA), which applies an anti-aliasing low-pass filter with a cutoff frequency of 50 Hz. EEG data were band-pass filtered with a passband frequency of 2 to 32 Hz.

#### Audio preprocessing

For both the stimulus reconstruction and binary classification methods, we pre-processed the two clean, audio streams to extract their broadband envelopes using the iterative algorithm in Horwitz-Martin *et al*.^30^. Envelopes were subsequently downsampled to a 100-Hz sampling rate.

### Linear decoding

To recreate the linear, stimulus-reconstruction approach in O’Sullivan *et al*.^13^ (see Fig. 1), we implemented a regularized, linear transform from EEG response data to audio envelope. We refer to the linear transform as LSQ, but in order to align the fitting of the linear model to the fitting of the neural network model in which the waveform is predicted, we used a Pearson correlation loss function instead of a mean squared error loss function.

The linear prediction of the audio waveform **y** is created with a simple matrix multiplication of the estimated weights, **w**, with a matrix of EEG data segments, **A**. Each row of **A** contains all the time points of the context window for all the EEG channels.

The LSQ weights, **w**, are often called the temporal response function (TRF) from the response-prediction EEG literature in which the EEG is seen as a response to the audio stimulus. Strictly speaking, when attention decoding is formulated in the backwards direction, the weights represent an inverse TRF.

The regularization parameter was selected on a per-subject, per-test-part basis from a set of ten logarithmically spaced values from 10^1^ to 10^10^. A robust standard scaling was applied to the training and testing audio and EEG data, also on a per-subject, per-test-part basis, using the estimated median and inter-quartile range of the training data. Each segment of data used for the LSQ method (and the DNN correlation-based method) was 26 samples long (approximately 250 ms given the 100-Hz sampling rate). Estimation was performed using Python 3 and Scikit-learn’s linear_model.RidgeCV method^31^. Internal cross validation was performed using a three fold split at the part level. Separate models were trained for each subject; no transfer learning across subjects was used in this analysis.

### Nonlinear decoding

The motivation for applying a deep neural network (DNN) to the AAD problem is that a non-linear decoder may provide improved performance relative to a linear decoder due to the inherent non-linear processing of acoustic signals along the auditory pathway. A DNN is a prototypical non-linear method flexible enough to handle multi-dimensional time series data. We use a neural network inspired by de Taillez *et al*.^23^ for the correlation-based classifier, and a novel convolutional DNN for the integrated classification decision architecture.

#### Neural network for stimulus reconstruction

**Figure 3:**
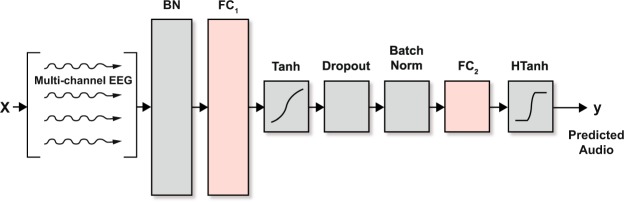
The neural network architecture for stimulus reconstruction, based on the design in de Taillez *et al*.^23^. There is one hidden layer with two nodes (FC_1_) to enforce significant compression of EEG data before being transformed to a predicted audio stimulus (see Fig. 1 for the system architecture). BN = batch normalization, FC = fully connected.

A simple neural-network architecture comprising a single hidden layer with two nodes was shown in de Taillez *et al*.^23^ to yield the best performance from a group of more complicated networks considered. Our adaptation of that network, shown in Fig. 3, includes batch normalization^32^ before the inputs to each layer, and a hard hyperbolic tangent (as opposed to a linear function) for the output layer’s activation to enforce our prior expectation that the audio envelope be bounded.

The network was trained with the Adam optimizer using a mini-batch size of 1024 samples, no weight decay, a learning rate of 10^−3^, and 2400 iterations. Following de Taillez *et al*.^23^ we also employed a correlation-based loss function rather than a mean-squared error-loss function to exploit the prior knowledge that we ultimately will be testing the reconstructed waveform and AAD performance with a correlation metric.

#### Neural network for direct classification

**Figure 4:**
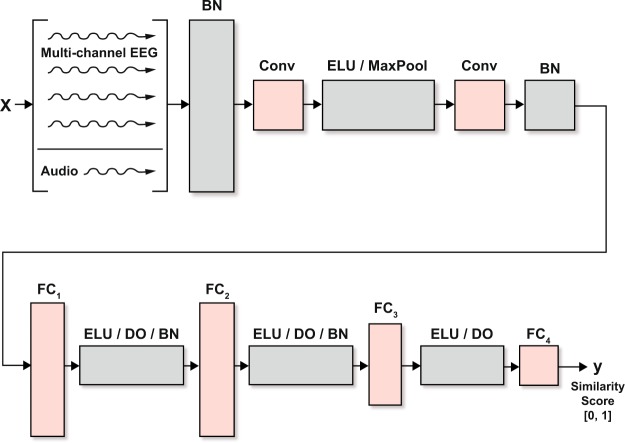
The convolutional architecture used for integrated similarity computation between EEG and a candidate audio stream. Components include batch normalization (BN), convolution layers (Conv_*i*_), exponential linear units (ELU), drop-outs (DO), and fully connected layers (FC_*i*_). Wet EEG (kernel, num ch in, num ch out): Conv_1_: 3 × 65 × 64, Conv_2_: 1 × 64 × 2, Dry EEG: Conv_1_: 3 × 19 × 19, Conv_2_: 1 × 19 × 2, Both: FC_1_: 246 × 200, FC_2_: 200 × 200, FC_3_: 200 × 100, FC_4_:100 × 1, MaxPool 1D, stride:2. See Fig. 2 for the system architecture.

Our novel end-to-end classification network with integrated similarity computation between EEG signals and a candidate audio envelope is pictured in Fig. 4. It comprises two convolutional layers, the first of which uses a kernel of three samples, and the second of which uses a kernel of one sample. The convolutional layers are followed by a set of four, fully connected layers that decrease in size in the later stages. We use batch normalization and dropout^33^ throughout, and the exponential linear unit^34^ for the non-linearity. Training includes a binary cross-entropy loss function, mini-batch size of 1024, Adam optimizer, no weight decay, and a learning rate of 10^−3^. We terminated the optimization process if the loss on the training set declined to below 0.09 or if the optimizer had run for 2400 steps. Because of computational limits on our computers, we randomly downsampled the 10-second set of samples over which a frame was evaluated by a factor of four.

### Methods of evaluation

#### Correlation-based evaluation

Algorithm performance was evaluated in a leave-one-out cross-validation paradigm across all audio parts presented to the subject. Multi-part training was performed by concatenating the presented audio data and recorded EEG response data. The concatenation was performed after each part was converted into a data matrix for the algorithm estimation to avoid discontinuities. The LSQ (linear) and DNN (non-linear) estimators were trained to reconstruct the attended audio using the training audio and EEG. Then, given the test EEG, each algorithm attempted to reconstruct the attended audio stimulus.

The estimated audio was then compared to the two candidate audio streams (attended and unattended) using Pearson correlation. The correlation was computed for ten-second, non-overlapping windows for the test part. If the left-out part was less than ten seconds, it was not evaluated. Decoding accuracy was computed as the percentage of 10-second windows for which the correlation coefficient with the attended audio envelope was higher than the correlation coefficient with the unattended audio envelope.

#### Classification-based evaluation

In the DNN classification architecture, the algorithm directly makes a similarity prediction between the recorded EEG and each of the candidate audio streams. In other words, the similarity metric is learned by the network during the training rather than dictated by the user. Given the similarity scores for each candidate audio stream, the attended stream is declared as the one with the highest score. To keep the decision rate the same between the two network architectures, we provide the classification algorithm data segments that are ten seconds in duration.

#### Computational environment

Analysis was performed using a mix of a GPU/CPU cluster and desktop computing environment running Python 3 and MATLAB (Mathworks, Natick, MA). The neural networks were implemented in PyTorch 1.0^35^, and parallelization across test folds was achieved with Nipype version 1.1.9^36^. The linear analysis used Scikit-learn version 0.19.1. An individual neural network train fold could be trained in less than a day.

#### EEG lead sub-sampling

The 64 channel wet EEG configuration contains a superset of the 18 channel dry EEG leads. As a third comparison between the systems, we sub-sampled the wet EEG leads to the dry subset.

## Results

### Decoding accuracy

**Figure 5:**
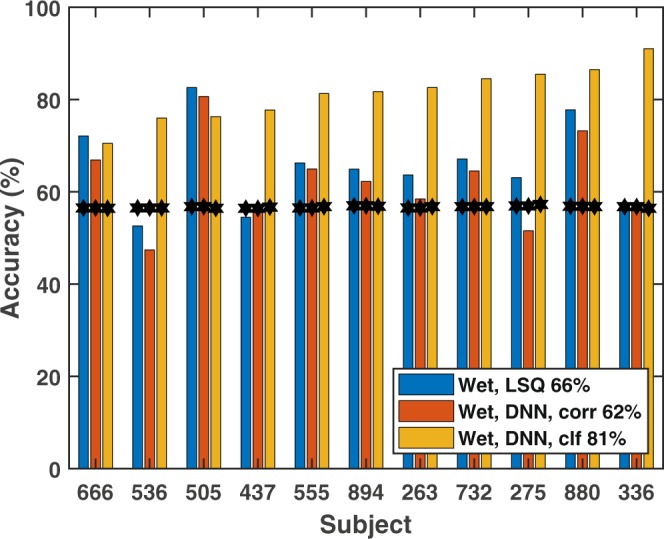
Per-subject attention-decoding accuracy using a wet, 64 channel EEG system. 10-second evaluation window, three algorithms: linear stimulus reconstruction (LSQ), non-linear stimulus reconstruction (DNN Corr.), and DNN classification (DNN Clf.). Chance performance is indicated by the black stars.

**Figure 6:**
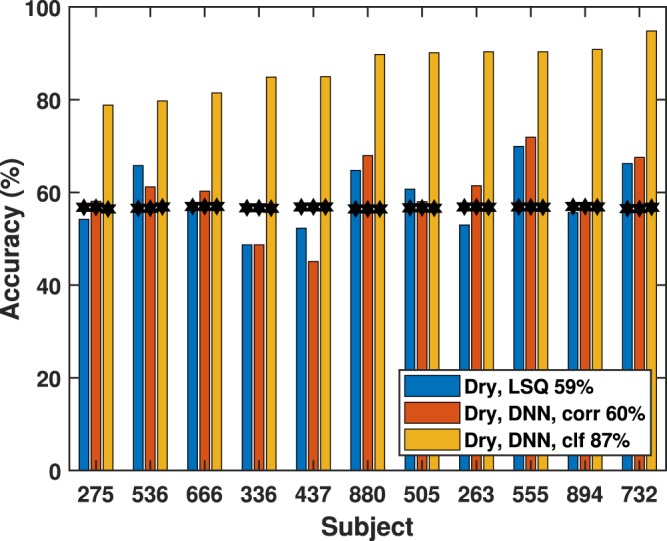
Per-subject attention-decoding accuracy using a dry EEG system. 10-second evaluation window, three algorithms: linear stimulus reconstruction (LSQ), non-linear stimulus reconstruction (DNN Corr.), and DNN classification (DNN Clf.). Chance performance is indicated by the black stars.

Decoding results for the wet EEG system are shown in Fig. 5, and for the dry EEG in Fig. 6. Each figure shows the per-subject average decoding accuracy using the linear correlation, neural-network based correlation, and DNN classification methods. Chance-level performance, indicated by the black stars, was computed as the 95^*th*^ percentile point of a binomial distribution with *p* = 0.5 and *n* equal to the number of non-overlapping 10-second windows. Mean decoding accuracies across subjects are summarized in Table 1, including those for which we sub-sampled the wet EEG channels to match the 18 channels (by location) of the dry EEG system for a more direct comparison of the two. A 2-way mixed-model ANOVA (EEG Type by Algorithm Type) was performed with subjects modeled as a random factor. We found a main effect for the choice of algorithm type (*F*(2, 80) = 144.0, *p* < 0.0001) but not for EEG type (*F*(2, 80) = 0.46, *p* = 0.64). The interaction between algorithm choice and EEG type was also significant (*F*(4, 80) = 2.8, *p* < 0.05). Tukey corrections were used for *post-hoc* multiple comparisons, and revealed statistically significant differences between the DNN classifier and both stimulus-reconstruction algorithms for both wet and dry EEG. There was no significant pairwise effect of the EEG type for any of three algorithms tested, including when sub-sampling the wet EEG channels to match the dry system (18 channels).

**Table 1:** Mean decoding accuracies for the three architectures and two EEG types.

	Stimulus Reconstruction		Classifier
	Linear	Nonlinear (DNN)	Nonlinear (DNN)
Wet EEG: 64 channels	66% (9%)	62% (10%)	81% (6%)
Wet EEG: 18 channels	63% (10%)	62% (8%)	85% (7%)
Dry EEG: 18 channels	59% (7%)	60% (8%)	87% (5%)

Standard deviations are shown in parentheses. The 18-channel wet EEG entries were computed using only the electrodes with positions that matched those of the 18 electrodes from the dry EEG cap.

### Visualization of LSQ TRF

**Figure 7:**
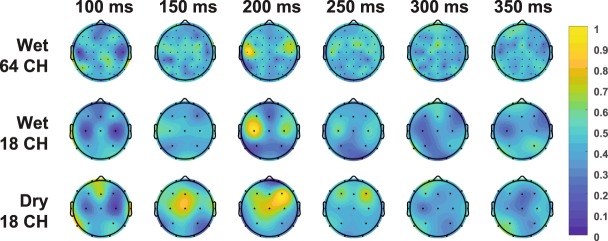
Normalized grand average headmaps of the LSQ TRF values across subjects.

For visualization, the linear kernel length was expanded from 26 to 51 samples in order to ensure capturing the full temporal evolution of the transform, but on average only the first half of the TRFs showed substantial non-zero activity. We normalize the TRF weights so the minimum weight is 0 and the maximum weight is 1. We compute the normalization separately for the wet and dry systems and per subject. Then, we average across the subjects and re-normalize again to a 0–1 for display as shown in Fig. 7 as a series of headmaps. We see a TRF peak occurs at 200 ms in the center of the head and dissipates afterwards. This timing is consistent with that reported previously where peaks near 200 ms also are shown^13,14,19^.

### Channel importance in the convolutional DNN

While obtaining insight into why a DNN performs as it does remains a challenging research question, we can gain some understanding of the convolutional DNN by examining the filter weights of the first convolutional layer. Essentially, this convolution is creating a set of linear combinations of the input EEG and audio channels. The full convolutional weight matrix is 3-dimensional (kernel by input channel by output channel), but we can collapse the 3D matrix into one dimension in order to visualize it. First, we select the middle element of the three-point temporal kernel, and then take the absolute value of the weights. Next, we sum the convolutional weights along the input channel. Taking the wet EEG as an example, there are 64 EEG channels and an audio channel as the input and 64 channels as the output from the first convolutional layer. As in visualization of the LSQ TRF, we normalize the 64 EEG weights of the 65-element vector so the minimum weight is 0 and the maximum weight is 1. We then apply that normalization to the 65^*th*^ audio element. We compute the normalization separately for the wet and dry systems and per subject. Then, we average across the subjects and re-normalize again to a 0–1 range using only the EEG weights but applying that normalization to the audio weight. Consequently, an audio weight greater than 1.0 is possible and signifies that audio is weighted more than any of the EEG leads.

**Figure 8:**
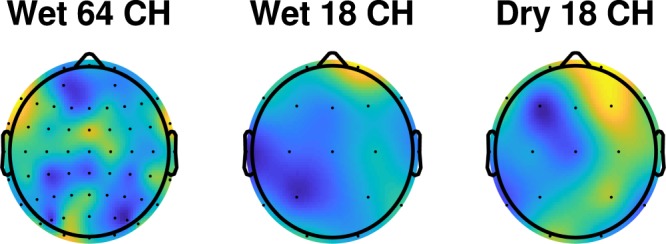
Normalized grand average headmaps of the mean convolutional weights for the wet, wet sub-sampled, and dry EEG systems for the DNN classifier network. The colors are scaled between 0 and 1. The audio channel weights (not shown) were 1.7, 1.1, and 0.77 for the wet, wet sub-sampled, and dry systems, respectively.

Figure 8 shows the mean absolute weights assigned to the wet, wet sub-sampled, and dry EEG datasets visualized as a headmap. Activated regions show some similarity to the LSQ TRF values in Fig. 7. Specifically, for the wet-EEG case, the central peak for the DNN headmap is roughly co-located with the 200 ms peak for the LSQ TRF. For the dry-EEG case, the elongated activation area to the right of the mid-sagittal plane resembles that for the 200 ms LSQ TRF (although the central peak at 200 ms is not evident in the DNN weights). The wet sub-sampled head map shows the same frontal activation strength as the dry map. Since the DNN classifier takes both audio (envelope) and EEG as an input, the audio channel should be weighted highly, and we see this is the case with the wet and wet sub-sampled EEG systems yielding audio weights of 1.7 and 1.1 and the dry electrode system yielding an audio weight of 0.77. This indicates that the network is utilizing both EEG and audio signals to make a decision.

## Discussion

As shown in Figs 5 and 6, and Table 1, both the nonlinear and linear approaches yielded comparable performance under the stimulus-reconstruction architecture. Decoding accuracy in our study varied more with the subject than with the choice of these algorithms. Typically, either both approaches performed well on a subject (*e*.*g*., Subj. 555), or both performed poorly (*e*.*g*., Subj. 437). The DNN classifier approach dramatically outperformed the traditional segregated architecture in decoding accuracy (81% wet, 87% dry) with a performance advantage in all of the dry EEG cases and all but two of the wet EEG cases, and shows a smaller variance among the subjects. While the exact reason for this is unclear, future work includes further analysis of the DNN’s weights to better understand its learned similarity metric. In addition, comparison of the DNN classifier to a logistic-regression classifier could yield insight into the importance of non-linearities in the decoding process.

In regard to the two EEG systems, overall decoding performance is comparable between the wet electrode and dry electrode systems. This result is somewhat surprising given that the wet system contains more than three times as many channels (64 vs. 18), although earlier work has shown a channel reduction from 96 to 20 had limited effect on decoding accuracy^14^. When we sub-sampled the number of wet electrode leads, we noticed a small increase in performance in the one-stage method suggesting some degree of overfitting was occurring with all 64 channels. Otherwise, the results did not change substantially. Further study is necessary to understand exactly how the various features of the two EEG systems (*e*.*g*., channel count, sensor type, choice of reference, etc.) interact and influence the decoding performance, although the successful decoding from both systems indicates that our novel DNN classifier approach is robust to different sensor configurations. Given these results, and recent studies that suggest that wet and dry EEG systems can deliver similar signal qualities (albeit with different systems than we used)^37^, a practical integration of AAD into an unobtrusive, wearable hearing device seems to be an achievable, long-term goal.

Of the three approaches we considered, two explicitly involve a backward model, *i*.*e*., stimulus reconstruction. We did not test the forward decoding architecture in this paper for both empirical and theoretical reasons. In regard to the former, the forward decoding approach has shown slightly worse performance than the backward decoding approach^20^. Theoretically, this performance loss is understandable because the auditory stimulus is just one of many internal and external factors, none of which is known other than the audio, that influence the corresponding EEG waveform. By contrast, because the neural activity represented in the EEG data is at least in part due to an auditory stimulus, it is reasonable to filter out the non-auditory components but retain the auditory component. As an extreme example, assume a model for the transform from audio to a specific EEG channel as the envelope of the audio plus additive noise, with the noise independent at each lead. In this case, the forward problem requires predicting noise, whereas the backward problem allows averaging out the noise across all the leads to recover the auditory envelope.

The performance of the linear approach in our study was lower than that reported in previous studies, potentially due to differences in the experimental design and decoding parameters. One significant difference between the results reported here and in other publications is that our talkers were co-located, *i*.*e*., combined digitally and delivered from a single loudspeaker in front of the subject. Reduced spatial separation (down to 10°) has been shown to have a detrimental effect on decoding accuracy in low (−1.1, −4.1, and −7.1 dB) but not high (20 dB) SNR conditions^18^, so it is not clear how strong an effect co-location had in this work. Other studies have included talkers at ±90° azimuth^13,15–18^, ±60°^19,20^, ±30°^14^, or ±10°^18^. We chose to use co-located talkers because this would provide a lower bound on decoding accuracy (from a spatial perspective) without extrapolating from an arbitrary separation angle.

A second potential reason for our relatively low linear decoding accuracy is that our correlation window (trial size) of 10 s and kernel length of 250 ms are shorter than those in some other experiments. Decoding accuracy previously has been shown to deteriorate with shortening trial sizes^17,20,38^, and one-minute^13,14^ and 30-second^16,18^ windows are more common in the literature. Our choice of 10 s was motivated by the fact that, a smaller window, eventually coupled with temporal smoothing such as that described in Miran *et al*.^24^, will be necessary for use with a practical, low-latency AAD system. Linear reconstruction kernels ranging from 250 ms^13,17^ to 500 ms^19,20^ have been reported, although no length has been shown to be optimal. We chose a 250 ms kernel based on early pilot data that did not indicate a significant improvement with an increase to 500 ms. Table 2 contains mean decoding accuracies for different correlation windows and kernel lengths to facilitate comparison to other AAD studies. Some improvement is seen with an increase in the correlation window length, but as with our pilot data, the kernel length had a negligible effect on performance.

**Table 2:** Mean decoding accuracy for the linear architecture with variations in the correlation window (10 s, 30 s) and the kernel size (250 ms, 500 ms).

	250 ms Kernel		500 ms Kernel	
	10 s Corr	30 s Corr	10 s Corr	30 s Corr
Wet EEG: 64 channels	66% (9%)	70% (12%)	63% (8%)	69% (13%)
Dry EEG: 18 channels	59% (7%)	63% (17%)	58% (7%)	62% (13%)

Standard deviations are shown in parentheses.

There are still several considerations in translating the decoding performance we are achieving to clinical utility. First, consistent with many other studies in the literature (Dau *et al*.^39^ is an exception), we focused on normal hearing listeners. We will need to recruit a substantial group of HI subjects to evaluate these algorithms for their use. Second, there is significant variance in decoding performance across individuals. In our study, participants were randomly prompted to repeat the last sentence from the attended talker, but the recall accuracy was consistently high and does not explain the variation in performance. In addition to traditional hearing loss, other potential factors that could affect AAD performance include cochlear synaptopathy, cognitive ability (*e*.*g*., working memory), and fatigue. Such factors have been considered in the context of the variability of traditional hearing-aid performance/acceptance^40^ and should be explored further in the context of AAD.

In conclusion, we have compared two different auditory-attention decision architectures, one which employs a Pearson based similarity metric to compare the reconstructed stimulus with actual stimuli (using a linear or DNN-based reconstruction approach), and a second, novel version in which the similarity transform is learned as part of the optimization process in a convolutional neural network. Furthermore, we evaluated all three algorithms with both a wet and a dry electrode EEG system using a two-talker AAD protocol. We found that the integrated decision-making architecture using a convolutional neural network yielded results comparable to state-of-the-art performance reported, and we have shown we can achieve this performance with both a wet and dry system where the talkers are not spatially separated. Future work includes validation on additional datasets to establish generalizablity, including evaluation of neural network architectures with around-the-ear^21^ and in-ear^22^ EEG electrodes. We also plan to employ transfer learning of network knowledge across subjects, and consider end-to-end neural network based architectures that combine both speaker separation and attention decoding, simply outputting the attended audio stream directly. This approach could be performed with single or multi-channel audio.

We plan to release both EEG datasets with baseline algorithms and benchmark performance metrics. We look forward to other research groups contributing their own analyses of this data in order to increase both the accuracy of decoding and shorten the latency of decoding. Improvements in both areas are needed for AAD to fulfill its promise as part of a complete, hearing-assistive system.

## Data Availability

The software is available from the corresponding author, and the dataset is available for collaborating institutions.
